# “OBE” Concept for New Training Mode of Electronic Information Science and Technology Professionals under Big Data Analysis

**DOI:** 10.1155/2022/8075708

**Published:** 2022-01-27

**Authors:** Jifeng Liang

**Affiliations:** Xi'an Fanyi University Engineering & Technology College, Xi'an 710100, China

## Abstract

As an educational concept based on learning output, OBE (Outcome-Based Education) is student-centered and emphasizes students' personal progress and learning achievement. Based on BD (big data) analysis, this paper proposes a new talent training mode for the electronic information science and technology specialty. This model employs BD analysis technology to examine the correlation index between social demand and talent cultivation, based on the employment situation of students in the country in previous years. The teaching reform was carried out under the OBE concept, and a new training scheme was formed. Training objectives, graduation requirements, curriculum system, and continuous improvement mechanism were all determined. This paper proposes an algorithm for determining the degree of similarity between the knowledge required by the organization and the knowledge held by its employees. The person with the highest similarity is identified as the training candidate by the algorithm, and the training candidate is then trained according to the knowledge that the organization requires. Talent training in the field of electronic information science and technology has yielded positive results.

## 1. Introduction

The development of a talent training scheme is a crucial link in university talent training, as well as a vehicle for innovating talent training modes and improving talent training quality [1]. The key to developing a professional talent training plan is to establish talent training objectives, such as professional employment positions, professional skills and qualities that graduates should possess, etc., to create a talent training objective system with specific characteristics, and then to develop a professional talent training plan to enable graduates to acquire corresponding employment skills. Current teaching methods place an excessive amount of emphasis on book knowledge mastery, neglect practical application of knowledge, and are unable to guide students' creative thinking through engineering cases. Traditional examinations continue to dominate the assessment method, while process evaluation and formative evaluation are still in their infancy.

OBE (Outcome-Based Education) is a kind of educational concept oriented to students' learning outcomes, which emphasizes that engineering education must focus on students' achievement of expected learning outcomes. In the whole OBE teaching concept, students are always the center of education and highly involved, and all teaching links are carried out around students. Guiding the reform of engineering education with the concept of OBE education is conducive to solving the problem of mismatch between the current graduates' abilities and social needs and has practical significance [2]. Engineering talents, as the internal driving force of social development, have a profound impact on China's scientific and technological development level and people's quality of life [3, 4]. Engineering talents trained by local engineering colleges are of vital significance to local economic development and national engineering construction. At present, scholars' research on talent training quality evaluation mainly focuses on talent training quality standard, evaluation subject, evaluation index system, evaluation mechanism, evaluation system, and so on and has achieved certain research results [5]. However, there are still many problems, such as limited access to evaluation information and lack of in-depth analysis due to the single evaluation method, the lack of scientific evaluation index system, the inefficacy of fairness and authority of evaluation results in practice, and the lack of objectivity of evaluation, which leads to low reliability, insignificant validity, and the difficulty of practical results [6, 7].

The performance evaluation and information construction of education have become the driving force for the development of current university education as a result of the information age's impact. However, as university teaching levels improve, BD (big data) analysis is required to scientifically evaluate teaching performance in the practice of the training mode of talents majoring in electronic information science and technology, and how to improve the scientificity and effectiveness of performance evaluation through the digital network platform is the focus of this paper. A new talent training model for electronic information science and technology is proposed in this paper based on BD analysis. A more perfect talent training scheme is designed to provide a more scientific training mode for the training and output of national high-end technical talents by analyzing subjective and objective influencing factors.

The creative point of this research is as follows: this paper adopts the technical platform of big data analysis, based on the theory of constructing the evaluation index system of talent cultivation quality suitable for the collection and analysis of big data evaluation model, and then collects and analyzes data through the construction of functional modules, taking a university as an example to get the evaluation results. After repeated several times, a relatively perfect big data evaluation model can be formed, which is more widely used in the evaluation of talent cultivation quality in universities.

## 2. Related Work

Literature [8] discusses in detail the origin, essence, system, and principles of foreign OBE educational ideas and preliminarily realizes the advantages of OBE educational ideas in personnel training. Literature [9] pointed out that the results-oriented education should be reflected in the process of making clear the teaching purpose, making the teaching plan, implementing, and evaluating it. OBE, as a good belief, atmosphere, and method, is worth learning and learning from higher education in China. Literature [10] tries to learn from the American results-oriented education model, help you understand its theoretical composition, formation process, and manifestation, and promote the localization process of results-oriented education by analyzing the feasibility of learning from China. Literature [11] points out as follows: in the future, the development of a flexible, high-level, and sustainable OBE education concept should be promoted. Literature [12] focuses on the analysis of the problems existing in the training mode of applied talents and advocates the introduction of OBE education concept into the training process of applied talents from three aspects: students' expected learning outcomes, assessment system of learning outcomes, and practical teaching system of applied talents. Literature [13] emphasizes that local engineering colleges should take various forms to contribute to local development, train high-quality undergraduate students for local industrial development, and give full play to their advantages to run several influential disciplines in China. Literature [14] mainly discusses and studies how to define the training orientation in local engineering colleges. This paper defines the local engineering colleges from four aspects: the purpose of running a school, the type level, the scale of running a school, and the development goal. Literature [15] puts forward the idea of applying OBE concept to the curriculum system reform of local engineering colleges, but the particularity of this subject of local engineering colleges is not clear in the specific suggestions.

One of a university's main functions is talent cultivation, and the quality of talent cultivation is the focus of the university's overall quality. It does not exist in isolation at the university but is influenced by a variety of other factors. It has a lot of connotations and can react to the university's talent cultivation, so more research on the quality of talent cultivation is needed. According to literature [16], our universities have first experienced the quality concept of knowledge, then the quality concept of ability, and finally the diversified quality concept since the founding of the People's Republic of China. According to literature [17], the quality concept of university talent cultivation should be appropriate to meet students' continuous growth. According to the literature [18], talent training quality must be evaluated from a multidimensional and three-dimensional perspective. According to literature [19], in the evaluation process, the relationship between the university and society should be strengthened, and the appropriate evaluation mechanism and indicators should be established as a result. Literature [20] attaches importance to the use value of talents, and puts forward that evaluating the quality of talent training should meet the needs of the country and society, and should meet certain standards in the degree of conformity. According to literature [21], this paper evaluates the quality of talent training in universities from three aspects: training conditions, training process, and training effect, using analytic hierarchy process. According to literature [22], the fuzzy comprehensive evaluation method in the management model is used to analyze the quality of talent cultivation in universities. Literature [23] divides the evaluation modes of Chinese universities from the philosophical evaluation theory, the stakeholder theory of economics, and the diversified concept of evaluation subjects of pedagogy and puts forward the strategies of authentication mode, ranking mode, and evaluation mode for the construction of university evaluation modes.

## 3. Research Method

### 3.1. Design of a New Training Mode for Electronic Information Science and Technology Professionals Based on BD Analysis

**Figure 1:**
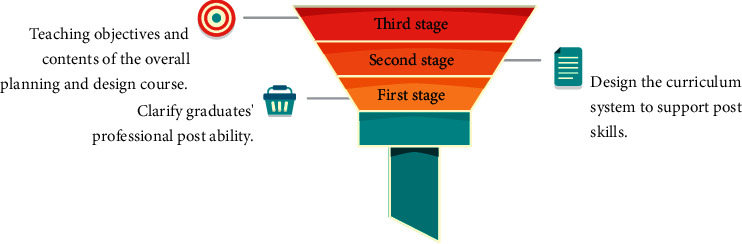
Three stages of personnel training of OBE concept.

OBE focuses on the training of professionals, which usually includes three stages (Figure 1).

The first step is to determine a graduate's professional postgraduation ability. To solve the problem of what kind of people the major trains, identify the target position of professional training, highlight professional characteristics, and analyze and refine the professional skills required by the major. The second step is to create a curriculum system that supports postsecondary skills. A reasonable curriculum system with ability expansion and extension is designed on the basis of clarifying job skills, guided by OBE education thought, focusing on the cultivation of students' ability literacy and quality literacy, in order to clarify the role of each course in achieving the training goal and solve problems such as why students should learn these contents. Third step is strong pertinence and adaptability in the overall planning and design of curriculum teaching objectives and teaching contents. The course teaching objectives are determined by the knowledge and skills needed to complete the work, in order to improve students' vocational skills and adaptability to the workplace, and to solve the problems of how to help students achieve these learning achievements, according to the needs of personnel training.

According to the training objectives and graduation requirements of computer electronic information science and technology specialty, combined with the general standard of engineering education certification and the supplementary standard of computer specialty, and according to the OBE education concept, the student-centered curriculum system is designed and constructed reversely.

**Figure 2:**
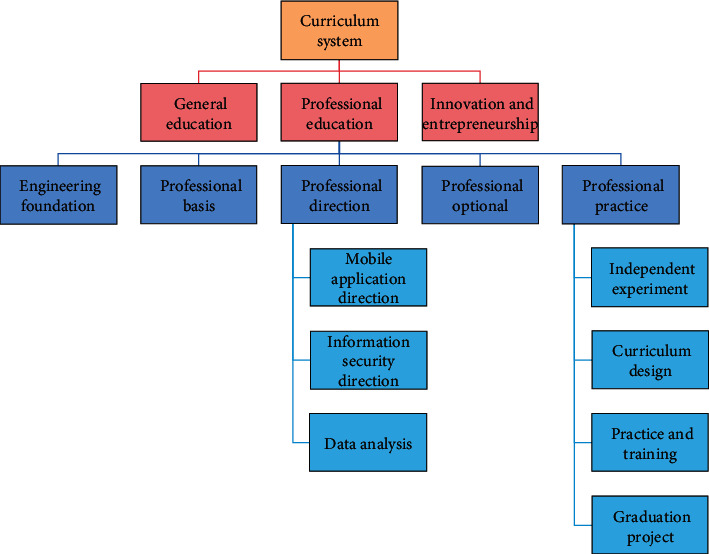
Curriculum system composition.

Corresponding to the graduation requirements, build a modular curriculum system which is oriented by ability training, pays attention to knowledge application and quality training, and embodies interdisciplinary integration. The curriculum system consists of general education, professional education, quality development, and innovation and entrepreneurship [24], as shown in Figure 2.

In Figure 2, the general education module courses are set according to the relevant national requirements, general standards and supplementary standards of computer, and electronic information science and technology specialty of engineering education certification.

The module of engineering foundation and specialty foundation must conform to the “National Standard for the Teaching Quality of Computer Science and Technology in Ordinary Colleges and Universities” formulated by the Teaching Steering Committee of the Ministry of Education and cultivate students' professional basic abilities such as computational thinking, program design and implementation, algorithm analysis and design, system ability, etc., which can solve practical problems [25].

The major's optional module includes a number of hot technical courses as well as professional knowledge development courses. Students can choose a number of courses that satisfy the credit requirements based on their personal interests and development goals. Artificial intelligence, pattern recognition, Linux system operation and maintenance, information retrieval, digital image processing, and other topics are among the topics covered. To improve the integration between teaching and employment, talent education and training programs should be integrated with practical occupations, a “dual” education mechanism should be built, and new teaching and training programs should be set up according to actual occupational needs. Simultaneously, schools and businesses must improve their communication and collaborate to develop new education and training courses that meet the needs of talent development and businesses.

The essence of personnel training is to enable personnel to master new knowledge. Therefore, after analyzing the above problems from the perspective of knowledge, it is found that the content of personnel training should be the knowledge needed by the organization, and the identified candidates should be the personnel who are closest to the knowledge needed by the organization. Therefore, the process of organizing personnel training can be divided into as follows.

Firstly, the knowledge needed by the organization is determined, then the personnel closest to the knowledge needed by the organization are identified as training candidates, and then the training candidates are trained according to the determined knowledge needed by the organization. According to the relationship between the knowledge network and the personnel network, the knowledge easily lost by the organization can be determined by quantitative calculation. On this basis, the areas easily lost by the organization can be determined, and then the knowledge needed by the organization can be determined according to the actual situation of the organization.

Easy-to-lose knowledge refers to the knowledge owned by very few people in the organization. With the loss of people, this knowledge is easy to lose. The judgment formula of easy-to-lose knowledge is as follows:

(1) $$\begin{array}{c} L\left(ke\right)=\left\{ke_{i}|\left|P\left(ke_{i}\right)\right|\leq \text{thr}\left(\text{elk}\right)\right\}. \end{array}$$

Among them, thr(elk) is the set threshold; when |*P*(*ke*_*i*_)| ≤ thr(elk), it is considered that *ke*_*i*_ is easy to lose knowledge. Ideally, thr(elk)=1. Easy-to-lose knowledge needs to be repaired and stabilized in time, and its judgment is helpful to recognize the weak links of organizational knowledge.

After clustering *L*(*ke*), a faction is formed, and the faction is regarded as a domain. Let *C*_*i*_(*K*) represent the set of knowledge elements that are easy to lose in domain *C*_*i*_ and judge whether *C*_*i*_ is a domain that is easy to lose according to the degree of domain knowledge loss.

The loss degree of domain knowledge is defined as follows: *C*_*i*_(*L*)/|*C*_*i*_|, where |*C*_*i*_(*L*)|, |*C*_*i*_|, respectively, represents the number of elements in their respective sets. Therefore, it means the proportion of the number of easily lost knowledge elements in *C*_*i*_ to the total number of knowledge elements. The judgment formula of easily lost domain can be expressed as follows:

(2) $$\begin{array}{c} Lc\left(C\right)=\left\{C_{i}|\frac{\left|\left|C_{i}\left(L\right)\right|\right|}{\left|C_{i}\right|}\geq \text{thr}\left(\text{eld}\right)\right\}. \end{array}$$

Among them, thr(elk) is the set threshold, and when |*C*_*i*_(*L*)|/|*C*_*i*_| ≥ thr(eld) is used, *C*_*i*_ is considered to be an easy-to-drain field, and ideally thr(eld)=100%.

To determine the knowledge that the organization requires, combine the knowledge that is easy to lose with the field that is easy to lose, and then combine the actual situation of the organization. Following the determination of the knowledge that the organization requires, the training candidates must be identified. The VSM (vector space model) is a traditional text-based calculation. The similarity between the organization needing knowledge and the personnel possessing knowledge represented by knowledge can be abstracted into the similarity between knowledge subjects if both the organization needing knowledge and the personnel possessing knowledge are regarded as knowledge subjects. Fields are formed after the knowledge network is clustered, and the similarity of knowledge subjects is investigated through the lens of fields.

In matrix *T*, rows represent knowledge subjects and columns represent fields, so the field vector of *P*_*i*_ is expressed as *T*_*i*_, so the Sim calculation based on the similarity between *P*_*i*_ and *P*_*j*_ of the field vector is shown in formula (3).

(3) $$\begin{array}{c} \text{Sim}\left(P_{i},P_{j}\right)=\cos\left({\vec{T}}_{\text{i}},{\vec{T}}_{\text{j}}\right)\\ =\frac{{\vec{T}}_{\text{i}}\cdot {\vec{T}}_{\text{j}}}{\left\|{\vec{T}}_{\text{i}}\right\|\cdot \left\|{\vec{T}}_{\text{j}}\right\|}\\ =\frac{\sum_{k=1}^{m}c_{ik}\times c_{ji}}{\sqrt{\left[\left(\sum_{k=1}^{m}c_{ik}^{2}\right)\sum_{k=1}^{m}c_{jk}^{2}\right]}}. \end{array}$$

When calculating the similarity, it needs to be calculated first according to the similarity algorithm based on the domain, then calculate it according to the similarity algorithm based on the VSM, and then make a comprehensive judgment according to the results. The similarity algorithm based on the VSM is a classic algorithm, with guaranteed accuracy and recall rate.

### 3.2. BD Evaluation Model of Talent Cultivation

The BD evaluation model includes two aspects in the evaluation of talent training quality: one is the theoretical basis, that is, the index system and the second is the technical route, that is, the functional module. In the process of construction, it is necessary to write the theoretical basis, that is, the evaluation index system, into the background operation module of the model, which is reflected in the compilation of the underlying code.

From an intuitive point of view, the formation process of the evaluation model is a process of “conditional input, analysis tools, analysis methods, output results, result interpretation, model test, model correction and improvement.”

**Figure 3:**
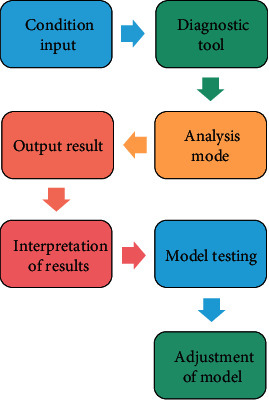
Flowchart of BD evaluation model construction.

In the process of building the concrete evaluation model, the main steps in the analysis mode are as follows: the first step, the acquisition scheme of evaluation data information, mainly through the crawler code to retrieve data and obtain evaluation information, and through the vertical search engine to trace the evaluation data layer by layer, obtain a certain amount of evaluation information and archive it to form the original database. The intuitive flow of BD model research is shown in Figure 3.

To improve the integration between teaching and employment, talent education and training programs should be integrated with practical occupations, a “dual” education mechanism should be built, and new teaching and training programs should be set up according to actual occupational needs. Simultaneously, schools and businesses must improve their communication and collaborate to develop new education and training courses that meet the needs of talent development and businesses. Then, according to the training results, the school conducts secondary theoretical training for students' shortcomings to realize the cooperation between classroom and practice, and enterprises should provide training bases for students to enhance their practical operation ability; finally, the school conducts secondary theoretical training for students' shortcomings to realize the cooperation between classroom and practice.

According to the above-mentioned teaching mechanism, the calculation formula of students' training investment index under the new mechanism is as follows:

(4) $$\begin{array}{c} p=\frac{\lambda \cdot \mu f\left(p\right)-\Delta f}{\sqrt{m}\cdot \left(b_{1}+b_{2}\right)}, \end{array}$$

where *p* represents the investment index; *λ* represents the correlation index between social demand and talent cultivation; *μ* represents the deterioration index when learning engagement is low; F *f*(*p*) represents a quantitative analysis function; Δ*f* represents analysis error; *m* represents the index change value; *b*_1_ said enterprise training investment parameters; and *b*_2_ indicates the impact index of the improved educational mechanism.

Rebuild the talent cultivation teaching system based on the obtained indicators, which includes developing a modular curriculum system between schools and businesses; establish a task-based teaching and training procedure; and create training programs that are multichannel, multilevel, and multicategory. To achieve a diversified and systematic teaching system, create a centralized task-oriented training mode. Students' achievement is an important standard to measure students' mastery of knowledge in the process of cultivating talents in the electronic information science and technology specialty. Students with abnormal learning status are identified through outlier algorithm analysis of their scores, and managers provide personalized guidance to these students. At the same time, the neural network model is used to predict the scores of key subjects for these students with abnormal learning status, and targeted learning is carried out for the subjects with problems.

The Adagrad algorithm can obtain small learning updates for nonsparse features according to different learning rates for each parameter, and on the contrary, obtain large learning updates. So, this optimization algorithm is more suitable for dealing with sparse feature data. For the Adagrad algorithm, the update iteration method is as follows:

(5) $$\begin{array}{c} \theta_{t=1,i}=\theta_{t,i}-\frac{n}{\sqrt{G_{t}+\varepsilon }}\cdot g_{t,i}, \end{array}$$

where *G*_*t*_ ∈ *R*^*dd*^ is a diagonal matrix, where diagonal element *e*^*ii*^ represents the sum of squares of the gradient of the *i*-th parameter *θ*_*i*_ from the past to the present, and *ε* is the smoothing parameter.

Adam (Adaptive Moment Estimation) is also a method to determine the gradient descent at different rates according to different parameters. Its calculation is different from the historical gradient attenuation, and the historical square attenuation is not used. Its attenuation mode is similar to momentum, as follows:

(6) $$\begin{array}{c} m_{t}=\beta_{1}m_{t-1}+\left(1-\beta_{1}\right)g_{t},\\ v_{t}=\beta_{2}v_{t-1}+\left(1-\beta_{2}g_{t}^{2}\right), \end{array}$$

*m*_*t*_, *v*_*t*_ is the weighted average and weighted deviation of gradient, respectively, and the initial value is 0 vector. Adam's researchers found that they are close to 0 vector, especially when the attenuation factor *β*_1_, *β*_2_ is close to 1. In order to solve this problem, the deviation of *m*_*t*_, *v*_*t*_ is corrected:

(7) $$\begin{array}{c} {\hat{m}}_{t}=\frac{m_{t}}{1-\beta_{1}^{t}},\\ {\hat{v}}_{t}=\frac{v_{t}}{1-\beta_{2}^{t}}. \end{array}$$

Finally, Adam's update equation is as follows:

(8) $$\begin{array}{c} \theta_{t+1}=\theta_{t}-\frac{\eta }{\sqrt{{\hat{v}}_{t}+\varepsilon }}{\hat{m}}_{t}. \end{array}$$

In order to know the students' learning situation and find out the problems in the teaching process in time, some courses adopt the formative evaluation method of unit test. Teachers can get timely feedback in the teaching process, adjust teaching plans, and improve teaching methods at any time. Finally, at the end of each course, a questionnaire survey was conducted on the achievement of the course objectives of the course *ε*.

## 4. Results Analysis and Discussion

### 4.1. Evaluation Results of Talent Training Quality

The above contents of this paper have completed the theoretical and technical construction of BD evaluation model. Here, a case study of the actual utility of this evaluation model is made, that is, the application of BD evaluation model. By applying the evaluation model to practical cases, the corresponding evaluation results are obtained, and the availability of the evaluation model is evaluated reasonably by analyzing the evaluation results.

**Figure 4:**
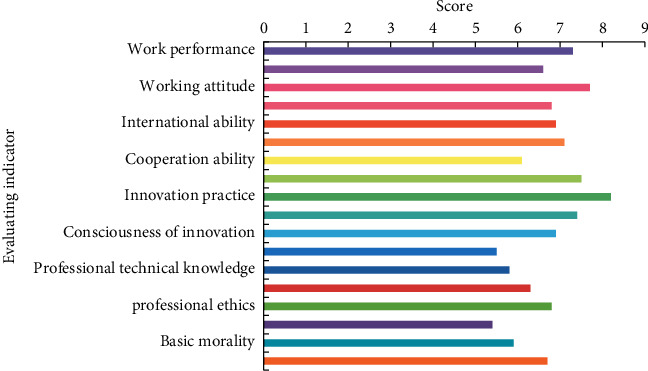
The evaluation index score of personnel training quality of employers.

On the evaluation of personnel training quality by employers, the index scores are shown in Figure 4.

Through the analysis of Figure 4, it can be concluded that in terms of fairness, due to the lack of BD collection technology and scoring dictionary in this study, there may be some errors with the real situation, so the results presented in this figure are only a reference and partly reflect the objective truth of the talent training quality of this school. In the interpretation of the diagram, based on the knowledge and understanding of the major of electronic information science and technology, the specific interpretation conclusions are as follows.

The four subindicators have little difference in scores among the ideological and moral indicators, but the gap from the full score is still not a high score. The index design of this study played a role in the formation of this result. Because this study focuses on evaluating graduates' employability, social adaptability is primarily reflected in aspects of knowledge, ability, and other characteristics that are easy to examine and quantify, resulting in less evaluation information in ideological and moral aspects. This school's students excelled in terms of creativity and practical ability. As a student at this school, I can see election notices and good news about various innovation competitions on campus based on my knowledge of the school. This school has put in a lot of effort to develop innovative students. In terms of social practice, the school is the only university in the area with a bachelor's degree or higher, and it has a significant influence in the community. As a result, a variety of businesses, communities, and other institutions and organizations are willing to collaborate with the school to organize social practice activities that will enrich the lives of college students. In terms of social ability, this school's students' international ability was praised and scored highly. The social evaluation data show that the students in this school have a good work attitude, but there are some areas where employers criticize them. For example, despite students' short-term job-hopping behavior, their overall performance in this school is above average. On the one hand, this achievement is due to the balanced dilution of data in statistics; on the other hand, it also reflects students' lack of adaptability after work, or a lack of career education in this field in university talent development.

**Figure 5:**
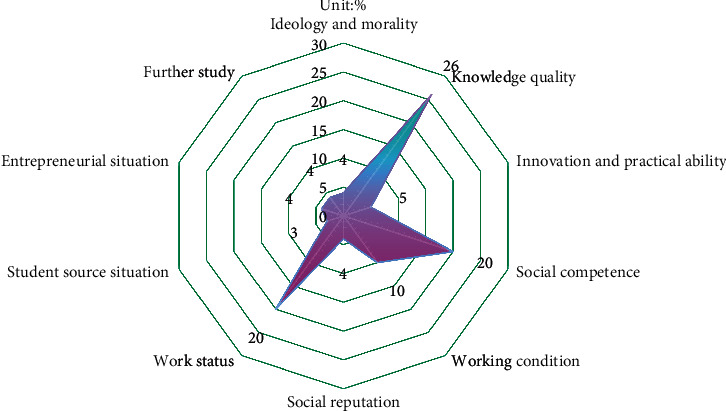
Proportion of indicators in importance.

Specifically, in this study, because in the setting of the index system, in addition to the connotation of the index, there is also the consideration of the index weight; here, the product obtained by multiplying the scores of ten first-level indexes by their weights indicates the importance of the index in the whole evaluation system, as shown in Figure 5.

Through the analysis of Figure 5, it can be known that in the process of evaluating the quality of the training of electronic information science and technology professionals, the graduates of this school have received the most attention in terms of knowledge quality, social ability, and employment status, while the network evaluation has not paid much attention to students' entrepreneurship, students' source, further study, and ideological status. This result also enlightens us that in the evaluation of talent training quality, pay attention to the employability and employment status of talents, and explore the evaluation of talent training quality from the specific connotation of these indicators.

**Figure 6:**
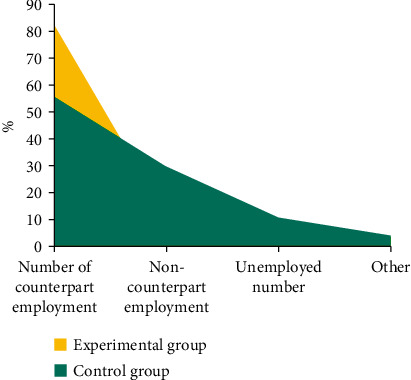
Test comparison results.

Take the employment statistics of students in the proposed talent training mode as the experimental group and the employment statistics of students in the traditional talent training mode as the control group.  Figure 6 shows the test results.

By analyzing the above two groups of test results, it can be seen that under the application of the talent training model of BD analysis, students can improve their own technical level by strengthening practical training ability. At the same time, because this mode adjusts the teaching mode in time according to the market demand for talents, it makes the students' professional skills match with the social needs, thus increasing the number of employed people.

However, students who study under the traditional talent training mode are not fully trained in practical courses, and do not dig out the characteristics of correlation indexes between society and talent technology, which leads to the deviation between students' personal skills and social needs, which leads to poor matching effect, and then affects students' employment.

### 4.2. Neural Network Model Predicts the Scores of Outlier Students

Before designing the neural network model, the data need to be further preprocessed. Organize the course scores, classify the course scores according to students, and cut out unnecessary data fields and some courses in the model design. Finally, the scores are processed, and the data are integrated according to three categories (the historical scores of the first five semesters of study courses, the name of target courses, and the category of target courses). When designing the neural network, the input layer inputs the scores of a certain student in the last semester, and the input dimension is 15. Finally, the probability of the category of the target course score is calculated through the Softmax layer. In the process of designing the neural network, three groups of experiments were designed. The number of neurons in each layer is shown in Table 1.

**Figure 7:**
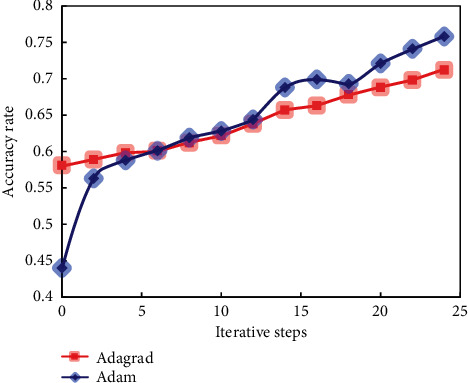
Algorithm comparison.

Through different optimizers, when the number of units in the first hidden layer is 10, the number of units in the second hidden layer is 5, and the number of units in the third hidden layer is 5, the accuracy of the classification results changes with the increase of iteration steps in the training process, as shown in Figure 7.

**Figure 8:**
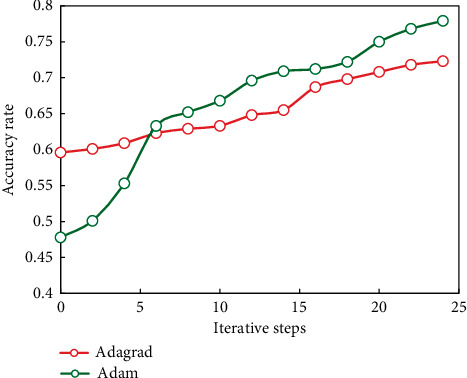
Algorithm comparison.

Similarly, when the number of the first hidden layer, the second hidden layer, and the third hidden layer are 50, 10, and 10, respectively, the change of the correct rate of the training set with the increase of the number of iterations is shown in Figure 8.

**Figure 9:**
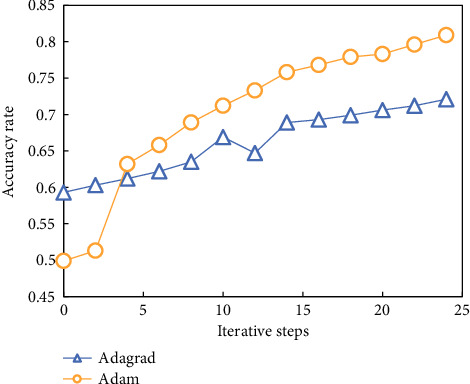
Algorithm comparison.

Similarly, when the number of units in the first hidden layer, the second hidden layer, and the third hidden layer is 100, 50, and 10, the number of iterations increases, and the change of accuracy rate is shown in Figure 9.

It can be seen from these figures that with the increase of iteration times, the correct rate of training is constantly increasing, and the increasing trend is basically caused by different optimization algorithms. It is found that the Adam algorithm is better than the Adagrad algorithm in the training process.

**Figure 10:**
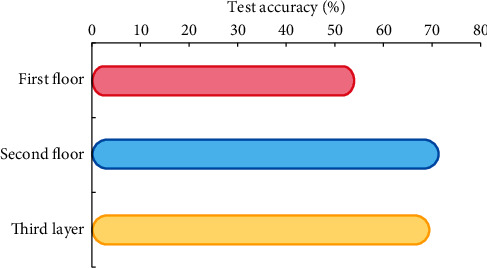
Test set accuracy rate.

When testing, the correct rate of Adam gradient update method is shown in Figure 10.

It is found that increasing the number of parameters will make the neural network model easier to over-fit, while too few parameters will make the model underfit. Finally, the Adam gradient updating method is used. The number of neurons in the first, second, and third hidden layers is 50, 10, and 10, respectively, as the final model.

## 5. Conclusion

The goal of OBE is to rebuild the curriculum system and update the curriculum training objectives to reflect changes in the industry, social needs, and the target orientation of skilled talent training. This paper examines the training model of electronic information science and technology professionals in universities with a BD background from various perspectives and performance evaluation frameworks, avoiding the blindness of university informatization decision-making, maximizing the scientific nature of large numbers, and laying a strong foundation for Chinese universities' modernization. In terms of data processing, the big data evaluation model is used to actively capture evaluation data on the network, and the importance of each index is scored using the emotion dictionary, after which the computer uses big data analysis technology to complete the comprehensive score of each index. Applying the OBE concept to BD technology and application talent training is a comprehensive reform of university talent training that also serves as a model for the training of professionals in electronic information science and technology.

The analysis of research status and specific application of OBE at home and abroad in this paper is not detailed enough, and some suggestions for improvement are too one-sided and general, and need to be further deepened and refined, due to the limited level of self-ability, which is limited by reference materials, number of articles, and writing time.

## Figures and Tables

**Table 1:** Setting of neurons in each layer.

Number of experimental groups	First hidden layer	Second hidden layer	Third hidden layer
1	10	5	5
2	50	10	10
3	100	50	10

## Data Availability

The data used to support the findings of this study are included within the article.
